# Identifying Key Factors in Papilla Growth Around Implants: Focus on Intraoral Negative Pressure

**DOI:** 10.3390/dj13030124

**Published:** 2025-03-13

**Authors:** Daniele Botticelli, Ivo Agabiti, Rihito Yamada, Nozomi Maniwa, Karol Alí Apaza Alccayhuaman, Yasushi Nakajima

**Affiliations:** 1 ARDEC Academy, 47923 Rimini, Italy; agabitiivo@gmail.com (I.A.); caroline7_k@hotmail.com (K.A.A.A.); y.nakajima@me.com (Y.N.); 2 Department of Oral Implantology, School of Dentistry, Osaka Dental University, 8-1 Kuzuhahanazonocho, Hirakata 573-1121, Osaka, Japan; rihito@yamadabr.com (R.Y.); nmani19850426@gmail.com (N.M.); 3 Department of Oral Biology, University Clinic of Dentistry, Medical University of Vienna, 1090 Vienna, Austria

**Keywords:** implant, mucosa growth, peri-implant mucosa, keratinized tissue, crown overcontour, BOPT, papilla growth

## Abstract

The absence of interdental papillae in dental prosthetics often leads to unsatisfactory esthetic outcomes, such as black triangles and elongated clinical crowns. While previous research has demonstrated that papillae can regenerate in a coronal direction, the underlying mechanisms remain incompletely understood. Several theories have been proposed to explain this phenomenon, but no clear cause–effect relationship has been established among the various factors involved in spontaneous papilla growth around implants. This study aims to identify and classify the factors influencing this process. Various potential contributors were analyzed, including adjacent elements, buccal–lingual papilla width, contact point position, convergent neck design, crown overcontour, intraoral negative pressure, and others. To systematically organize these factors, a modified Overton Window and a mind map were employed. The factors were categorized as cause-related, essential, or influencing based on the collective opinion of the research group following a comprehensive review of the relevant literature. In the absence of clear evidence supporting a definitive cause–effect relationship, Occam’s Razor (the principle of parsimony) was applied to identify the most plausible cause-related factors.

## 1. Introduction

In dental prosthetic treatments, an unsatisfactory esthetic result frequently arises from the absence of interdental papillae, leading to the appearance of black triangles and the pronounced elongation of clinical crowns due to the recession of marginal soft tissues. Our earlier research demonstrated that gingival papillae and tissue margins are capable of naturally regenerating in a coronal direction [1,2]. Some theories have been proposed to explain this phenomenon, such as the inflammatory [3] and the intraoral negative pressure theories [1,2,4]. Moreover, several conditions have been identified as crucial for the occurrence of the phenomenon such as the existence of a sufficient band of crestal keratinized mucosa, both in terms of width and thickness [1,2,5,6,7]; the presence and positioning of the contact point [8,9,10]; an appropriate distance between adjacent teeth or implants [11,12]; an ideal bucco-lingual dimension of the alveolar ridge (crestal bucco-lingual thickness) [10]; and, lastly, the availability of spaces around the prostheses that can be occupied by the growth of crestal soft tissues [1,2,4,13].

Histologically, the interdental papilla consists of dense connective tissue covered by masticatory mucosa and oral epithelium, with its shape influenced by the position of contact points, interproximal width, and the underlying alveolar crest [14]. Soft tissue adaptation around implants is highly dependent on collagen remodeling, fibroblast activity, and extracellular matrix dynamics. Following tissue injury, resident cells activate signaling pathways that regulate proliferation, differentiation, and migration into the wound site [15]. During the early healing phase, fibroblasts drive collagen synthesis and matrix deposition, while myofibroblasts emerge in the remodeling phase to orchestrate wound contraction [15,16]. This balance between matrix remodeling and myofibroblast activity determines the extent of creeping attachment, which is critical for papilla regrowth. If wound contraction is excessive, fibrosis and scarring may occur, impeding tissue flexibility and limiting soft tissue adaptation [17,18].

Beyond cellular and molecular processes, mechanical and functional factors significantly influence soft tissue growth [16]. The periosteum plays a crucial role as a reservoir of regenerative cells and provides a structural template for guided tissue regeneration. Its cambium layer contains osteoprogenitor cells, while its vascular network supplies essential growth factors for tissue remodeling [19]. Importantly, the use of a partial-thickness flap has been shown to influence periosteal activation and facilitate tissue adaptation [20]. By preserving the periosteum and allowing lateral repositioning, this surgical technique enhances vascular supply and regenerative potential, promoting controlled soft tissue migration [21,22]. Clinical evidence suggests that this approach, in combination with biomaterials and space-maintaining techniques, can optimize papilla preservation and coronal soft tissue displacement. Studies have demonstrated that soft tissue grafting procedures contribute to improved peri-implant conditions by increasing mucosal thickness and keratinized mucosa, enhancing bleeding indices, significantly reducing probing depth, and preserving higher marginal bone levels [23,24,25]. However, similar improvements in peri-implant conditions have also been observed in the absence of soft tissue grafting [1,2]. This suggests that additional factors may contribute to these outcomes, as mechanical forces generated by mastication and functional movements have been shown to influence fibroblast behavior and extracellular matrix organization [26]. Among these forces, intraoral negative pressure, generated during activities such as swallowing and speaking [27,28,29,30,31,32,33], has been hypothesized to contribute to soft tissue migration by creating subtle suction forces that may facilitate coronal displacement of the papilla [1,2,13]. This functional factor, although less studied than surgical and biological determinants, warrants further investigation as a potential modulator of spontaneous soft tissue migration.

In scientific research, the exploration of unresolved problems, such the spontaneous papilla growth, often leads to the formulation of multiple competing theories. Each theory offers a potential explanation, yet not all are equally valid or plausible. Determining cause–effect relationships in research is crucial because it allows researchers to identify the underlying mechanisms that drive observed phenomena, ensuring that actions performed by clinicians are based on a clear understanding of how specific factors influence outcomes. In the absence of a clear causal relationship, determining which theory most accurately reflects reality requires the application of certain principles that guide scientific methodology.

Hence, due to the lack of a clear cause–effect relationship among the various factors involved in the spontaneous papilla growth around implants, this article aims to identify and classify the most important factors influencing this process.

## 2. Factors Evaluation and Classification

### 2.1. Study Design and Assessments

The analysis was conducted by five independent expert clinicians, each with over 10 years of experience in implant dentistry, oral surgery, and/or prosthodontics. These evaluators were selected for their extensive expertise and dedication to long-term patient care, which provided valuable insights into evaluating clinical outcomes. Each clinician was affiliated with either a private practice or a clinical university setting, ensuring an unbiased and comprehensive assessment process. During the initial kick-off meeting, the clinical problem was presented, and relevant literature was discussed and distributed for further review.

### 2.2. Tools Used for Evaluation

Mind Map—A mind map, introduced by Tony Buzan, is a visual thinking tool de-signed to organize information in a way that mirrors the brain’s natural associative processes. It starts with a central concept, from which branches radiate outward, representing related ideas or subtopics. These branches can further divide into sub-branches, breaking complex ideas into simpler, interconnected components. This structure enhances com-prehension, memory, and creativity by engaging both the logical and creative aspects of the mind through the use of colors, keywords, and images. Mind maps are particularly effective for brainstorming, planning, and learning [34,35].

Overton Window—The Overton Window is a concept from political science that de-scribes the range of ideas or policies considered acceptable or mainstream within public discourse at a given time. Named after policy analyst Joseph Overton, the window represents the spectrum of what is politically feasible. Ideas outside this range are often deemed too radical or extreme. As public opinion shifts, the window can move, allowing previously unthinkable ideas to become discussable, acceptable, and eventually widely adopted. This framework is valuable for understanding how societal norms evolve and how policymakers and advocates can influence public perception over time [36].

Occam’s Razor—Occam’s Razor, attributed to the medieval philosopher William of Ockham (c. 1287–1347), is a heuristic principle in scientific methodology. Also known as the principle of parsimony, it suggests that when faced with competing hypotheses that explain a phenomenon equally well, the simplest explanation—requiring the fewest assumptions—should be preferred [37]. This principle is rooted in the notion that unnecessary complexity may introduce additional sources of error or uncertainty, potentially obscuring understanding. While not a definitive rule, Occam’s Razor is a valuable tool for guiding hypothesis selection, promoting clarity and efficiency in scientific explanations. It emphasizes a logical, streamlined approach that aligns with observed evidence.

### 2.3. Methods Applied for Evaluation

Key factors were first defined, and four categories were identified. Analytical tools from other fields were selected to support the study. Each factor, along with the four identified categories, was organized into a table, and assessors were asked to assign each factor to a specific category.

Each factor was evaluated for its role in papilla growth, starting with an assessment of any potential cause–effect relationship. The analysis then determined whether the factor played an essential role in papilla growth or merely influenced the outcome. Factors showing both a positive causal relationship and consistency with a healthy papilla were retained within their respective categories. Conversely, factors with a positive causal relationship but not aligned with a healthy papilla were placed in a filtered-out group. Factors without a detected causal relationship were classified as either essential or influential, depending on whether their presence was necessary or merely contributory.

**Figure 1:**
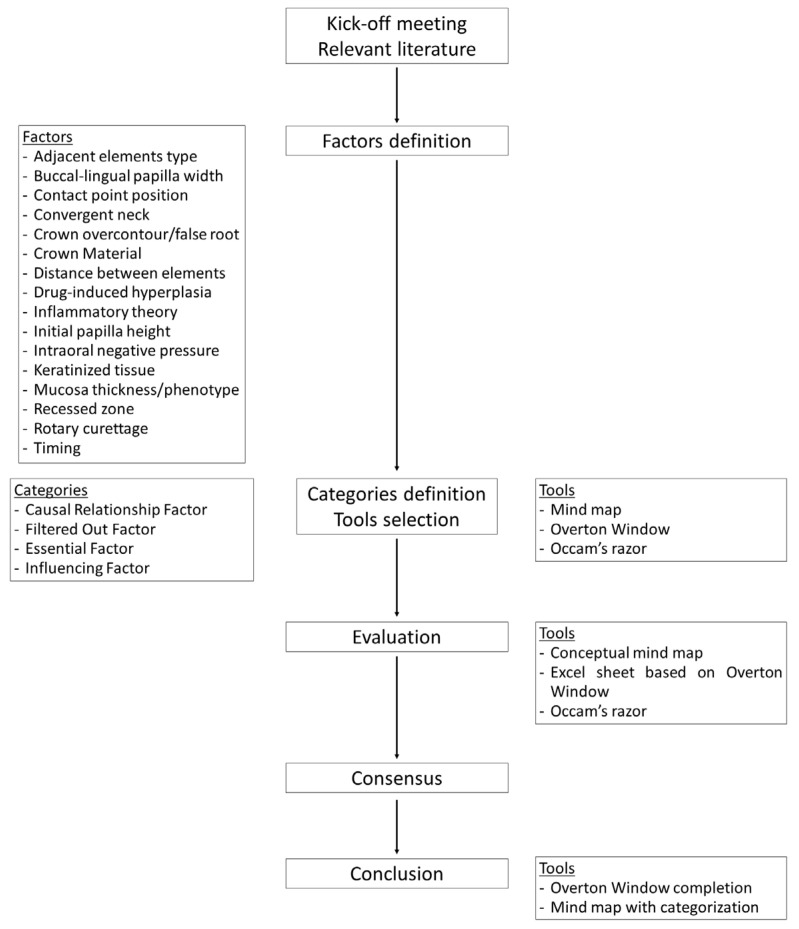
Flowchart illustrating the steps for the classification of the factors involved in the spontaneous papilla growth.

During the consensus meeting, the results of these assessments were reviewed and discussed for each factor. In cases of diverging evaluations, the panel conducted a thorough discussion to reach a consensus, guided by the available scientific evidence and rational analysis (Figure 1).

### 2.4. Categories Applied

- These categories were applied to each factor for consistency with the reported scientific evidence, together with a rational analysis:
- Causal Relationship Factor—Identified when a potential cause-and-effect relationship with papilla growth is observed.
- Filtered Out Factor—Factors that exhibit a cause-and-effect relationship with papilla growth but whose effects are attributable to inflammation or drug influences and, therefore, should not be associated with healthy tissue. This classification may also be applied to factors that are neither essential nor influential.
- Essential Factor—Determined as critical for papilla growth. Papilla growth cannot occur in the absence of this factor, despite no direct cause-and-effect relationship being observed.
- Influencing Factor—Recognized as having an impact on papilla growth, although not essential for it. No direct cause-and-effect relationship is observed with papilla growth.

### 2.5. Factors Evaluated

Factors were organized in a table in alphabetical order, without prior grouping, to allow for an unbiased evaluation based on the adopted classification criteria. Key articles related to each topic were included to provide foundational references. The principle of Occam’s Razor was applied during the categorization process to ensure simplicity and avoid unnecessary complexity in assigning factors to their respective categories.

After evaluation, at the consensus meeting the following inferences were drawn:

- Adjacent elements type: No cause-related relationship was found. The nature of the neighboring elements was considered to influence papilla growth [1,9].
- Buccal-lingual papilla width: No cause-related relationship was found. The buccal-lingual width was considered to influence papilla growth [10].
- Contact point position: No cause-related relationship was found. The contact point position was considered essential for papilla growth [8,9,10,38].
- Convergent neck: No cause-related relationship was found. The convergent neck was considered to influence papilla growth [39,40,41,42].
- Crown material: No cause-related relationship was found. The material seems not to have a relevant effect on marginal soft tissue growth. Consequently, this may be regarded as a factor that might influence papilla growth [1,42].
- Crown overcontour/false root: No cause-related relationship was found. The overcontour or the presence of a false root were considered as influencing papilla growth [1,2,13,42].
- Distance between elements: No cause-related relationship was found. The distance between elements was considered essential for papilla growth [9,11,12].
- Drug-induced hyperplasia: Drug-induced hyperplasia presented a causal relationship with papilla growth. However, hyperplastic condition was not considered a healthy condition [43,44,45].
- Inflammatory theory: The inflammatory theory presented a causal relationship with papilla growth. However, an inflammatory condition that causes edema and inflammatory infiltrate, leading to tissue growth, cannot be considered healthy [3]. The biofilm theory was invoked primarily in the context of orthodontic treatment, given the noticeable papilla growth despite the absence of significant plaque accumulation [46].
- Initial papilla height: A post hoc analysis of data from a previously published study growth [1] revealed that a lower initial height of the papilla was associated with a greater increase in papilla score (see Table S1). No cause-related relationship was found. This factor was considered to influence papilla growth.
- Intraoral negative pressure: The intraoral negative pressure has been measured in several studies [27,28,29,30,31,32,33] and suggested as a possible causal relationship with papilla growth [1,2,4].
- Keratinized tissue: No cause-related relationship was found. The presence of keratinized tissue was considered essential for papilla growth [1,2].
- Mucosa thickness/phenotype: No cause-related relationship was found. The mucosa thickness was considered to influence papilla growth [5,6,7].
- Recessed zone: No cause-related relationship was found. The presence of a recess zone was considered essential because it provides space for papilla growth [1,2,13].
- Rotary curettage: No cause-related relationship was found. In implant therapy, this condition is generally absent, unless we consider the surgical trauma during abutment connection as relevant. At most, this trauma may be regarded as a factor that potentially influences papilla growth [42,47].
- Timing: No cause-related relationship was found. The timing was considered to influence papilla growth [1].

**Figure 2:**
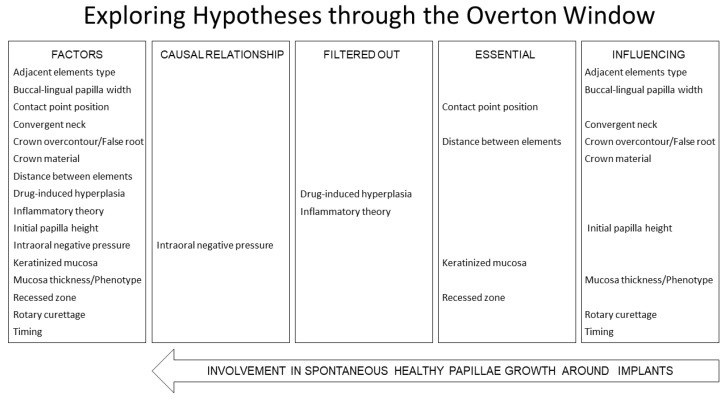
A modified customized version of the Overton Windows.

The results were summarized and presented using a modified Overton Window (Figure 2).

**Figure 3:**
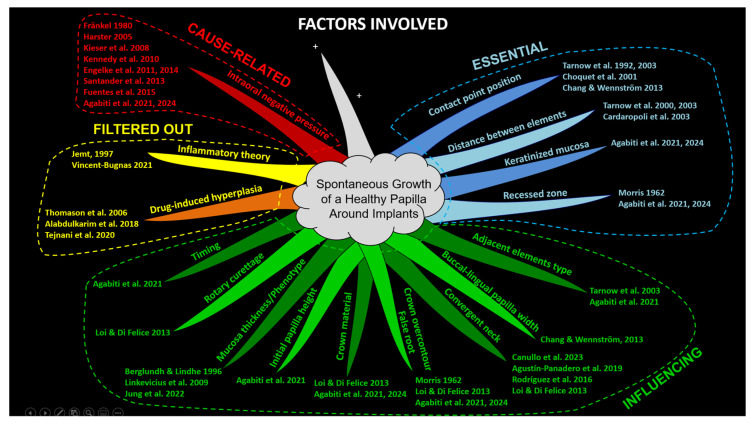
The factors were organized into four distinct categories, each identified by different colors and boxed outlines to enhance visual clarity and differentiation between the categories. The white branch leaves room for additional factors that may still be identified. The ‘+’ symbol is used to indicate placeholders for new factors and their respective citations. Note that only main branches were used to enhance the map’s visual clarity and to highlight flexibility in categorization [1,2,3,4,5,6,7,8,9,10,11,12,13,27,28,29,30,31,32,33,38,39,40,41,42,43,44,45,46,47].

Finally, after the evaluation performed through the Overton Window, a mind map (Tony Buzan) was prepared to allow a better visualization of all groups of factors (Figure 3).

## 3. Discussion

A well-defined cause–effect relationship for the spontaneous papilla growth has not yet been established. Multiple factors contribute to this process, and in this article, we aimed to identify the most relevant among them and clarify their respective roles. We categorized these factors into three groups: essential factors, which are necessary for papilla growth to occur; influencing factors, which may affect the process but are not mandatory for its initiation; and cause-related factors, which may have a direct effect on papilla growth. An essential factor is defined as one whose absence would result in no or minimal papilla growth. In contrast, influencing factors can modulate the extent or quality of growth but are not strictly required for it to take place. Finally, cause-related factors are those that actively trigger the mechanisms necessary to stimulate papilla growth.

This study highlights intraoral negative pressure as a potential cause-related factor among those evaluated. Unlike other factors, it involves an active mechanism—namely, a suction effect generated by intraoral negative pressure—which may play a direct role in the growth process. However, this does not imply that it is definitively the cause; it simply represents a plausible explanation after excluding other possibilities. It is important to note that excluding other potential causes does not confirm the remaining factor as the definitive cause. A direct cause–effect relationship still needs to be conclusively demonstrated, and in the case of the spontaneous papilla growth, such a relationship has not yet been proven.

It is important to highlight that intraoral negative pressure is generated during swallowing [27,28,29,30,31,32,33] and persists until the lips are opened [32,48]. Although this pressure is lower in intensity, it lasts longer than the brief negative pressure created during swallowing. Given that a person swallows about 200–1000 times per day (mean frequency 585 per day) [49], this low-intensity negative pressure may exert its influence over extended periods, potentially playing a role in papilla growth. When prosthetic crowns are designed with an over-contoured profile in the apical third, they inherently create recessed areas around the collar. This results in local anatomies that resemble or even exaggerate the natural enamel–cement junction. In such scenarios, the lips, cheeks, and tongue press against the most prominent sections of the crowns and alveolar ridges, forming an underlying chamber around the collar. This area has been previously described as an inaccessible area by Morris [13] and later referred to as an inaccessible space by Agabiti [1]. The intraoral negative pressure functions as an “oral pump” [4,28], potentially promoting gradual papilla growth into a recessed zone [1,2,4]. In other words, the effect of negative pressure on the papilla might occur through a suction mechanism that operates in small doses over time during each swallowing process, gradually stimulating tissue growth. This low-intensity negative pressure is fundamentally different from the acute suction effect observed in cupping therapy—an ancient alternative medicine technique that uses suction cups on the skin, purportedly to relieve pain and inflammation and improve blood flow [50,51].

The present study identified intraoral negative pressure as the most plausible cause of papilla growth around implants, after excluding other factors such as inflammation and drug-induced hyperplasia using the principle of parsimony. While this assumption offers a promising direction, it is important to emphasize that a direct cause–effect relationship has not yet been conclusively demonstrated. Furthermore, many factors influencing papilla growth around implants may also apply to tissue growth around teeth, particularly in the context of the Biologically Oriented Preparation Technique (BOPT) principle [42].

To understand the research approach employed, it may be helpful to analyze the three types of inferential reasoning proposed by Charles Sanders Peirce (1839–1914): deduction, induction, and abduction [52,53].

Deduction is an inferential process that starts with general premises and leads to specific conclusions. It requires an initial rule, which forms the basis for predictable results, heavily depending on the reliability of this rule. Deductive reasoning is deeply integrated into everyday routines, from expecting the alarm to ring at a set time, to turning on the hot water faucet and anticipating hot water to flow. In clinical practice, we similarly rely on deduction, for example, when placing a dental implant in the alveolar bone, expecting osseointegration based on established principles, or performing specific treatments with predictable outcomes.

Induction, on the other hand, involves generating rules through experimentation. In the absence of pre-existing rules, inductive methods are employed by conducting experiments or clinical research. For instance, introducing a new variable to a test group while maintaining a control group under standard conditions enables the comparison of outcomes. This process helps identify significant differences and establish new rules, which can later be used in deductive reasoning.

In the case of spontaneous papilla growth, the exact cause remains unclear. Under such conditions, we start by forming conjectures or hypotheses about possible reasons for this growth, with negative pressure being one of several potential causes identified. This type of reasoning aligns with what Peirce referred to as abduction—the inferential process of forming a plausible hypothesis based on a set of observations. Abduction, often described as “the best possible explanation”, plays a crucial role in generating new ideas and theories. While it does not guarantee the truth of its conclusions or even their probability, it provides an initial explanation that can be further tested through induction. Abduction serves as the foundation of the scientific inquiry process, enabling the hypothesis to be tested and refined. This abductive reasoning was the process applied in this study, supported by selected analytical tools to identify a potential cause-related factor.

When multiple variables and factors are involved, as in the case of spontaneous papilla growth, tools are necessary to assist in the problem-solving process. The modified Overton Window applied in this study identified intraoral negative pressure as ‘the best possible explanation’ for spontaneous papilla growth [36], while inflammation and drug-induced hyperplasia were excluded as they are not associated with a healthy papilla.

The principle of parsimony, often referred to as Occam’s Razor, was instrumental both in these exclusions and in the categorization process [37]. This logical approach prioritizes the simplest explanations that are consistent with the observed evidence. When multiple theories can explain the same phenomenon, the simplest one—with the fewest assumptions—is preferred. While parsimony does not guarantee that the simplest theory is correct, it serves as a heuristic to guide scientists in choosing between otherwise equally plausible explanations [37]. By applying this principle, we excluded inflammation and drug-induced hyperplasia as less likely causes, thereby narrowing the focus to the most plausible cause-related relationship. This approach also proved valuable in classifying factors—albeit subjectively—as either essential or influencing, thereby streamlining the evaluation process.

Other tools employed in this problem-solving study included the conceptual map in the form of a mind map. The conceptual map visually organized all relevant information, clarifying relationships between ideas and enhancing understanding of a complex topic like spontaneous papilla growth. It provided a framework that encouraged critical thinking by visually structuring the information. Mind maps, popularized by Tony Buzan, are designed to reflect the brain’s natural thinking process, promoting free-flowing, non-linear thought and fostering creativity [34,35]. In this study, the mind map helped break down the complex topic of spontaneous papilla growth into simpler, visual representations, allowing simultaneous comprehension of both the big picture and detailed components. The mind map incorporated Gestalt principles such as similarity, where elements with similar attributes (e.g., color, shape, size) are perceived as part of the same group or pattern and proximity, where elements located close to one another are perceived as related or grouped together [54].

**Figure 4:**
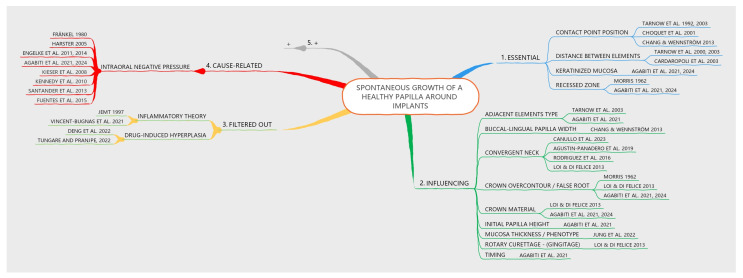
Mind map with sub-branches, generated using a specialized software (EdrawMind v. 12.0.4, Edrawsoft, Shenzhen, China). The grey branch leaves room for additional factors that may still be identified. The ‘+’ symbol is used to indicate placeholders for new factors and their respective citations [1,2,3,4,5,6,7,8,9,10,11,12,13,27,28,29,30,31,32,33,38,39,40,41,42,43,44,45,46,47].

One branch of the mind map was intentionally left open to indicate that additional factors, not yet considered, may be added by the readers. The version of the mind map presented in the article was intentionally simplified to include only main branches, omitting sub-branches. However, an alternative version is shown in Figure 4, which incorporates main branches as well as sub-branches at different levels. This representation was created using one of the many available software tools that facilitate quick and efficient mind mapping (EdrawMind v. 12.0.4, Edrawsoft, Shenzhen, China).

Both mind maps were designed to adhere to Tony Buzan’s original principles while providing flexibility. Their structure allows for easy reassignment of factors to different categories, accommodating alternative interpretations or preferences that readers may have regarding the authors’ initial categorizations.

In this article, we discussed papilla growth, acknowledging that some may argue a papilla adjacent to an implant surface should not be considered a true papilla due to significant histological differences compared to periodontal tissue [55]. However, from a clinical perspective, these differences are not readily discernible. Therefore, we continue to use the term “papilla” to describe the keratinized tissue filling the embrasures between elements.

When multiple factors appear to play a role in spontaneous papilla growth, yet no definitive data exist to determine their individual contributions, we can only assess their potential influence and formulate hypotheses. According to Peirce [52,53], hypothesis generation is based on abductive reasoning. To support this reasoning, we have attempted to organize the various factors involved by employing analytical tools from non-biological fields.

The Overton window and Buzan’s mind mapping approach serve as useful tools for systematically identifying and prioritizing the key factors involved. Although the tools applied in this study do not provide direct biological explanations, they serve to identify potential cause-related factors that future research can investigate to either confirm or refute the theory proposed.

A recently published article demonstrated that shaping the crown to create “inaccessible” spaces not only facilitates papilla growth but also promotes the formation of keratinized tissue on the buccal aspect, thereby addressing esthetic concerns [1]. While existing data on the dimensions of these spaces remain incomplete, the present study provides a comprehensive analysis of the essential and influencing factors that must be considered for the development of both papilla and keratinized tissue around implant-supported crowns.

Traditionally, the essential factors taken into account include the presence of keratinized tissue, the distance between the contact point and the bone crest, and the interproximal spacing. However, one critical factor has not been adequately considered: the recess zone. To create this “inaccessible space”, prosthetic crowns and pontics should be designed with an over-contoured profile at the buccal apical third, forming an under-contoured recessed area at the collar that mimics a false root [1,2,13]. This recessed space may play a crucial role in facilitating the action of intraoral negative pressure, which could stimulate the growth of both keratinized tissue and the papilla around the crown.

While the findings highlight intraoral negative pressure as a potential driving factor, spontaneous papilla growth is inherently governed by multiple interconnected factors. Despite the fact that this negative pressure has been documented for 150 years [56], no one has yet attempted to demonstrate a causal relationship with spontaneous papilla growth. Negative pressure has been considered primarily in the context of swallowing but has rarely been explored in relation to the growth of intraoral tissues. This, combined with the challenge of developing an experimental system capable of accurately evaluating a causal relationship, has hindered progress in research on this topic. Therefore, it would be useful and interesting to develop a device capable of experimentally applying this negative pressure to assess its potential effects on the growth of intraoral keratinized tissues. Research should prioritize controlled clinical trials to empirically test the hypothesis of intraoral negative pressure as a driver of papilla growth. Studies could also investigate the interplay of multiple factors, such as the combined effects of intraoral pressure, keratinized tissue presence, and contact point positioning, as well as genetic predisposition, systemic health, and patient habits that may influence papilla regeneration. Further exploration of the biological mechanisms underlying this phenomenon, potentially through histological studies comparing papilla formation under various conditions, would be highly valuable. Expanding the scope to include diverse patient populations and varying implant types could provide more generalized and comprehensive insight.

The main limitation of this study is its reliance on theoretical analysis and existing literature, which may not fully capture the complexity of spontaneous papilla growth. Additionally, the classification of factors using the modified Overton Window [36] and the principle of parsimony (Occam’s Razor) [37] involves an inherent degree of subjectivity, which may introduce bias. Moreover, the study did not include experimental or clinical trials to validate the proposed relationships, limiting the ability to draw definitive conclusions. It should also be noted that this study did not aim to provide a systematic review of the available literature on the topics discussed. Instead, we selected a range of well-known articles, prioritizing those that offered relevant data for each factor under consideration and were essential to achieving the study’s objectives. As part of the mind map we used, we intentionally left one branch open to encourage readers to explore additional factors they deem significant, using the Overton Window framework for classification.

## Data Availability

The data are available following a reasonable request.

## Supplementary Materials

The following supporting information can be downloaded at: https://www.mdpi.com/article/10.3390/dj13030124/s1, Table S1.
