# Supplementary material for: Identifying Key Factors in Papilla Growth Around Implants: Focus on Intraoral Negative Pressure
Source: Dent J (Basel). 2025 Mar 13;13(3):124. doi: 10.3390/dj13030124 (PMC11940915; doi:10.3390/dj13030124)
Supplement: Supplementary file 1 [file dentistry-13-00124-s001.zip › dentistry-3468873-supplementary.pdf]

Post-hoc analysis of data from a previously published study on papilla growth [1]. This analysis applying score according to Jemt classification [2], revealed that a lower initial height of the papilla was associated with a greater increase in papilla height.			
Mesial aspect		Distal aspect	
Initial score	Gain at the follow-up	Initial score	Gain at the follow-up
0	2.1	0	1.8
1	1.0	1	1.0
2	0.5	2	0.7

1. Agabiti I, Apaza Alccayhuaman KA, Nakajima Y, Botticelli D. An enigmatic soft tissue creeping phenomenon: The spontaneous peri-implant mucosa margin and papilla growth. A retrospective clinical study. Clin Exp Dent Res. 2021 Aug;7(4):474-483. doi: 10.1002/cre2.380. Epub 2020 Dec 21. PMID: 33350149; PMCID: PMC8404504.

2. Jemt T. Regeneration of gingival papillae after single-implant treatment. Int J Periodontics Restorative Dent. 1997 Aug;17(4):326-33.
